# Piperlongumine, a Novel TrxR1 Inhibitor, Induces Apoptosis in Hepatocellular Carcinoma Cells by ROS-Mediated ER Stress

**DOI:** 10.3389/fphar.2019.01180

**Published:** 2019-10-14

**Authors:** Qianqian Zhang, Weiqian Chen, Xiuling Lv, Qiaoyou Weng, Minjiang Chen, Ri Cui, Guang Liang, Jiansong Ji

**Affiliations:** ^1^Key Laboratory of Imaging Diagnosis and Minimally Invasive Intervention Research, the Fifth Affiliated Hospital of Wenzhou Medical University, Affiliated Lishui Hospital of Zhejiang University, Lishui Central Hospital, Lishui, China; ^2^Chemical Biology Research Center, School of Pharmaceutical Sciences, Wenzhou Medical University, Wenzhou, China

**Keywords:** thioredoxin reductase 1, reactive oxygen species, hepatocellular carcinoma, endoplasmic reticulum stress, piperlongumine

## Abstract

Hepatocellular carcinoma (HCC) is the sixth most common cancer and the third leading cause of cancer-related deaths globally. Despite advances in diagnosis and treatment, the incidence and mortality of HCC continue to rise. Piperlongumine (PL), an alkaloid isolated from the fruit of the long pepper, is known to selectively kill tumor tissues while sparing their normal counterparts. However, the killing effects of PL on HCC and the underlying mechanism of PL are not clear. We report that PL may interact with thioredoxin reductase 1 (TrxR1), an important selenocysteine (Sec)-containing antioxidant enzyme, and induce reactive oxygen species (ROS)-mediated apoptosis in HCC cells. Our results suggest that PL induces a lethal endoplasmic reticulum (ER) stress response in HCC cells by targeting TrxR1 and increasing intracellular ROS levels. Notably, PL treatment reduces TrxR1 activity and tumor cell burden *in vivo*. Additionally, TrxR1 is significantly upregulated in existing HCC databases and available HCC clinical specimens. Taken together, these results suggest PL as a novel anticancer candidate for the treatment of HCC. More importantly, this study reveals that TrxR1 might be an effective target in treating HCC.

## Introduction

Liver cancer, the sixth most common human malignancy and the third leading cause of cancer mortality, is a major public health problem, and hepatocellular carcinoma (HCC) represents more than 90% of primary liver cancers (Zhou et al., 2017). Typically, HCC is usually diagnosed at an advanced stage, and many patients with advanced stage HCC are not eligible for curative therapies (Liu et al., 2015a). Moreover, the effects of traditional systemic chemotherapy on HCC and the survival rate are poor (Del Pozo and Lopez, 2007; Cidon, 2017). Thus, the identification of a novel therapeutic approach for treating HCC is urgently needed.

**Figure 1:**
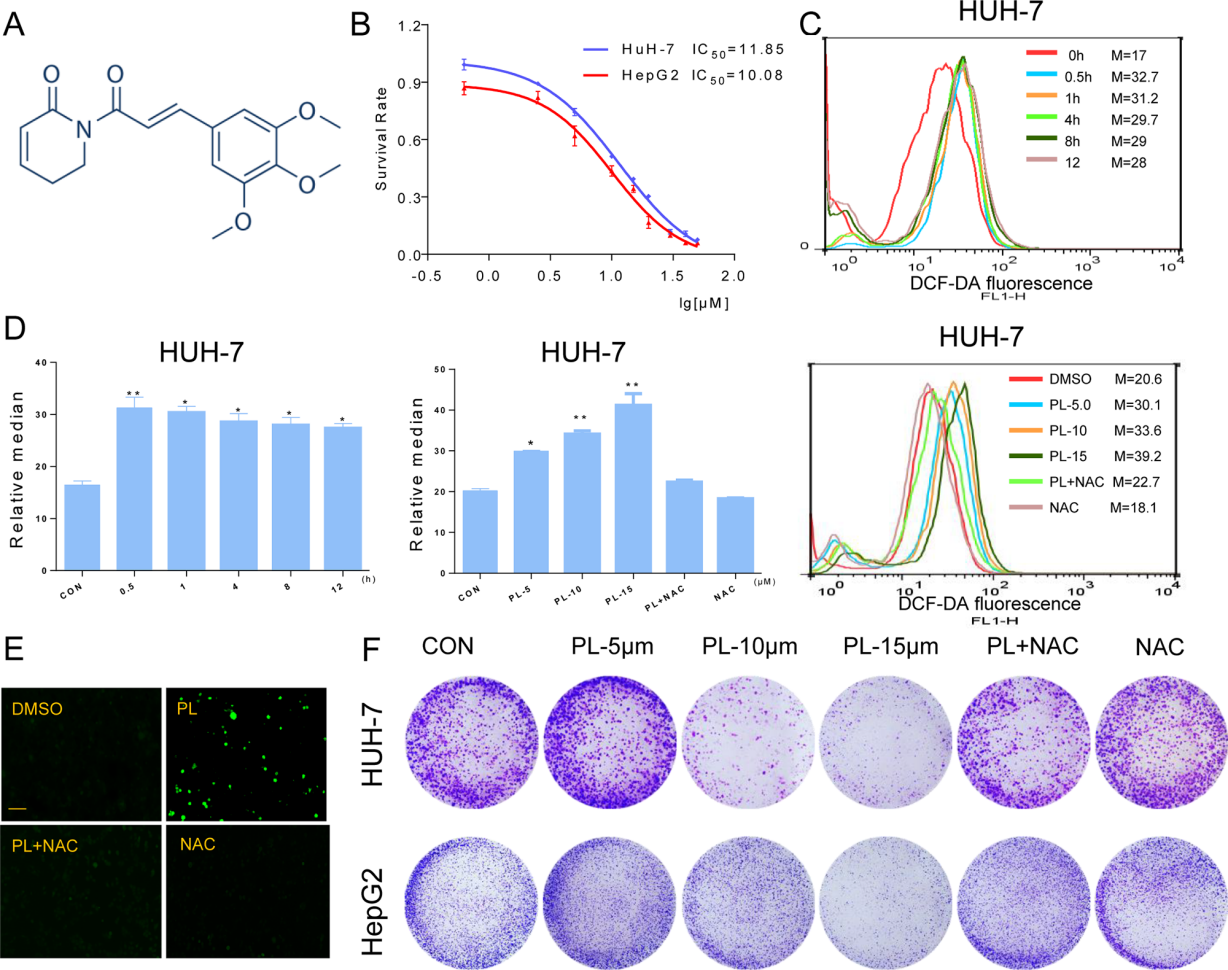
PL inhibits cell growth and induces ROS accumulation in HCC cells. **(A)** Chemical structure of PL. **(B)** The effect of PL on the proliferation of HCC cells. Cells were incubated with increasing doses of PL for 24 h, respectively. Cell viability was determined by MTT assay. **(C)** Intracellular ROS generation in HUH-7 cells was determined in a time- and dose-dependent manner using the redox-sensitive dye DCFH-DA (10 μM). HUH-7 cells were treated with PL (15 μM) for the indicated times. HUH-7 cells were preincubated with or without 5 mM NAC for 2 h before exposure to PL at the indicated concentrations for 30 min. Intracellular ROS generation was measured by flow cytometry. **(D)** Quantification of 2’-,7’dichlorofluorescein (DCF) fluorescence data from **(C)**. **(E)** Intracellular ROS generation induced by PL was measured by fluorescence microscopy. Magnification, 200×. Bar, 100 µm. HUH-7 cells were preincubated with or without 5 mM NAC for 2 h before exposure to PL (15 μM) for 30 min. Then, intracellular ROS generation was measured by fluorescence microscopy. **(F)** Effect of PL treatment on colony formation. Cells were preincubated with or without 5 μM NAC for 1 h before exposure to PL at the indicated concentration for 5 h and then stained with crystal violet on day 8. Data represent similar results from three independent experiments. Error bars represent the S.E.M. of triplicate experiments (*p < 0.05, **p < 0.01).

Piperlongumine (PL) is a naturally occurring small molecule derived from the fruits and roots of the long pepper plant (Karki et al., 2017). The chemical structure of PL has been well characterized (**Figure 1A**). PL has been used in traditional Ayurvedic medicine to treat gastrointestinal and respiratory diseases for a thousand years (Xiong et al., 2015). Recent studies demonstrated that PL is highly and selectively toxic toward cancer cells, strongly suggesting that PL is a promising bioactive agent for liver cancer therapy (Gong et al., 2014). PL has been proposed to induce cancer-selective cell death by elevating reactive oxygen species (ROS) levels (Jin et al., 2014). However, the mechanism by which PL induces ROS remains poorly defined, and the primary cellular target and mode of action of PL in HCC are still unclear.

Continuous oxidative stress resulting from the generation of ROS by environmental factors or cellular mitochondrial dysfunction has recently been associated with the progression of HCC (Sosa et al., 2013). Simply put, ROS play a key role in the development of cancer. A moderate increase in ROS can promote cell proliferation and differentiation, whereas excessive amounts of ROS can cause oxidative damage to crucial cellular macromolecules and lead to cell death (Cairns et al., 2011; Taniguchi et al., 2012; Glasauer and Chandel, 2014). Therefore, modulating ROS homeostasis or oxidative stress-responses has been proposed as an effective therapeutic strategy for cancer.

The thioredoxin (Trx) system, which consists of NADPH, Trx reductase (TrxR), and Trx, is a vital antioxidant system that plays a critical role in regulating cellular redox processes (Lu and Holmgren, 2014; Koharyova and Kollarova, 2015). There are three mammals TrxR1 (H-TrxR) isoforms: TrxR1, which is found in the cytoplasm; TrxR2, which is found in mitochondria; and TrxR3 (also called Trx glutathione reductase, TGR), which is expressed only in specialized tissues (e.g., the testis) (Arner, 2009; Rigobello and Bindoli, 2010). TrxR1 is overexpressed in many human tumors and has emerged as a valuable target for anticancer drug development (Mahmood et al., 2013; Fan et al., 2014). Generally, one possible mechanism is that cancer cells atypically drive their reductive pathways to maintain cell viability and escape from the cytotoxic effects of increased ROS. The Trx system using NADPH channeled through Trx reductase 1 (TrxR1) is one of the major redox systems. Mounting evidence suggests that many redox regulators are involved in resistance to anticancer drugs (Arner, 2017). Targeting TrxR1 has been shown to occur with many different electrophiles with anticancer potential (Cebula et al., 2015).

In the present study, we noticed that TrxR1 is overexpressed in clinical liver HCC and that PL could inhibit TrxR1 activity to induce oxidative stress in HCC. Additionally, PL could induce apoptotic cell death in HCC cells *via* activating the ROS-dependent endoplasmic reticulum (ER) stress pathway. Taken together, our findings provided a molecular mechanism by which PL kills liver cancer cells and shed light on how PL works *in vivo*.

## Materials and Methods

### Reagents

PL (S7551) was purchased from Selleck Chemical (Shanghai, China), the purity of PL is 99.33%. N-Acetylcysteine (NAC) was purchased from Sigma-Aldrich (St. Louis, MO, USA). PI was purchased from BD Pharmingen (Franklin Lakes, NJ). Hoechst stain and DCFH-DA were purchased from Beyotime Biotechnology (Nantong, China). Anti-Cdc2, anti-Bcl-2, anti-Bax, anti-CyclinB1, anti-TrxR1, and anti-Ki67 antibodies were purchased from Santa Cruz Biotechnology (Santa Cruz, CA), and anti-ATF-4, anti-EIF2α, anti-CHOP, and anticleaved caspase-3 antibodies were purchased from Cell Signaling Technology (Danvers, MA). HRP-conjugated secondary antibodies were also obtained from Cell Signaling Technology.

### Cell Culture

Human HCC cell lines (HUH-7 and HepG2) were purchased from the Institute of Biochemistry and Cell Biology, Chinese Academy of Sciences (Shanghai, China). HUH-7 cells were cultured in DMEM medium (Gibco, Eggenstein, Germany), whereas HepG2 was grown in MEM(Gibco). All medium formulations were supplemented with 10% heat-inactivated fetal bovine serum (Gibco, Eggenstein, Germany), and cells were grown in a humidified cell incubator with an atmosphere of 5% CO_2_ at 37°C.

### Cell Viability Assay

HUH-7 and HepG2 cells were seeded into 96-well plates at a density of 8 × 10^3^ cells per well in DMEM and MEM, respectively, containing 10% heat-inactivated FBS for 24 h and allowed to attach overnight. PL was dissolved in DMSO and diluted with DMEM or MEM to final concentrations of 0.625, 2.5, 5, 10, 15, 20, 30, 40, and 50 µM. The cells were incubated with PL for 24 h before the (Mosman, 1983) MTT assay.

### Determination of Cellular ROS

Cellular ROS generation was measured by flow cytometry. Briefly, 5 × 10^5^ cells were plated in 6-well culture dishes and allowed to attach overnight. The cells were then treated with PL at different concentrations and for different indicated times. Then, the cells were stained with 10 μM DCFH-DA (Beyotime Biotechnology, Nantong, China) at 37°C for 30 min. The cells were harvested and then washed three times with ice-cold PBS, and fluorescence was measured by flow cytometry (FACSCalibur, BD Biosciences, CA). In some experiments, the cells were pretreated with 5 mM NAC for 2 h. In all experiments, 8,000 viable cells were analyzed.

### Colony Formation Assay

Cells were seeded in 6-well plates at 500 cells per well for 24 h and then preincubated with or without NAC for 1 h before PL treatment for 5 h. One week later, the cells were stained with a crystal violet solution (0.5 crystal violet in 25% methanol) to assess colony growth.

### Determination of Morphological Features of Apoptosis

A total of 5 × 10^5^ cells were plated on 60-mm dishes, allowed to attach overnight, and then treated with PL (15 μM) in the presence or absence of NAC (5 mM) for 2 h. Twenty-four hours later, the cells were fixed, washed twice with PBS, and stained with Hoechst or PI or acridine orange and ethidium bromide staining solution according to the manufacturer’s instructions. The cells were observed and imaged using a fluorescent microscope (Nikon, Tokyo, Japan) with 20× amplification.

### Cell Transfection for Gene Silencing

ATF4 and TrxR1 siRNA oligonucleotides were synthesized by GenePharma (Shanghai, China). HUH-7 cells were seeded at a density of 1 × 10^5^ in 6-well plates for 24 h. siRNA against human ATF4, TrxR1, or nontargeting control siRNA (GenePharma) were transfected at a final concentration of 50 pmol (ATF4: sense 5’-GCCUAGGUCUCUUAGAUGATT-3’; antisense 5’-UCAUCUAAGAGACCUAGGCTT-3’) or 100 nmol (TrxR1: sense 5’-GCAAGACUCUCGAAAUUAUTT-3’; antisence 5’-AUAAUUUCGAGAGUCUUGCAG-3) using lipofectamine 3,000 reagent (Invitrogen, CA) in serum-free medium for 6 h. Complete growth medium was then added and the cells were cultured for an additional 24 h. Levels of silenced genes were determined by western blotting and apoptotic cell death was assessed by acridine orange and ethidium bromide dual staining.

### Cell Cycle Analysis

Cells were placed on 60-mm plates for 24 h and then treated with PL (5, 10, or 15 μM) for 16 h in the presence or absence of NAC (5 mM). The cells were then collected and centrifuged at 1,000 rpm for 5 min. The supernatant was discarded, and the isolated cells were washed with ice-cold PBS. After being resuspended in 100 µL PBS, the cells were fixed with ice-cold 75% ethanol and stored at −20°C for 12 h. After centrifugation, the cells were washed twice with ice-cold PBS and then stained with PI at 4°C for 20 min in the dark. Cell cycle analysis was performed with a FACSCalibur flow cytometer. The fractions of cells in G2/M phase were used for statistical analysis using FlowJo 7.6 software (TreeStar, San Carlos, CA, USA).

### Western Blot Analysis

Cells or tumor tissues were homogenized in protein lysis buffer, and debris was removed by centrifugation at 12,000 rpm for 10 min at 4°C. Protein concentrations in all samples were quantified by using a Bradford protein assay (Bio-Rad, Hercules, CA). Protein samples were separated using 6–12% sodium dodecyl sulfate-polyacrylamide gels and transferred to PVDF membranes. The blots were blocked for 2 h at room temperature with freshly prepared 5% nonfat milk in TBST. Blots were then probed with specific primary antibodies overnight at 4°C. Horseradish peroxidase-conjugated secondary antibodies and an ECL kit (Bio-Rad) were used for protein detection.

### Endpoint Insulin Reduction Analysis

Untreated HUH-7 cells or xenografted tissues were collected and lysed with RIPA buffer in the presence of protease inhibitors. The concentrations of protein in the cell lysate and tumor tissue lysate were determined using the Bradford method. Cell extracts containing 50 μg of total proteins were incubated in final reaction volumes of 50 μl containing 4 μM E. coli Trx, 0.4 mM NADPH, and 0.32 mM insulin for 30 min at 37°C. Then, the reaction mixtures were incubated at room temperature for 2 h. The reactions were terminated by the addition of 100 μl of 1 mM DTNB in 6 M guanidine hydrochloride (pH 8.0), and the absorbance at 412 nm was measured using a microplate reader. The blank value was subtracted from the corresponding absorbance value of the sample. The activity was expressed as the percentage of the control.

### Hepatoma Xenograft Model

All animal experiments complied with Wenzhou Medical University’s Policy on the Care and Use of Laboratory Animals. Protocols for animal studies were approved by the Wenzhou Medical College Animal Policy and Welfare Committee (approved documents: 2016/APWC/0046). Five-week-old athymic BALB/c nu/nu female mice (17–20 g) were purchased from Vital River Laboratories (Beijing, China). The mice were housed at a constant room temperature with a 12/12 h light/dark cycle, fed a standard rodent diet, and given water ad libitum. The mice were blindly and randomly divided into two experimental groups. A total of 5×10^6^ HUH-7 cells in 100 μl of PBS were subcutaneously injected into the right flank of the mice. When tumors reached a volume of 50–100 mm^3^, the experimental group was treated with intraperitoneal injections of PL (10 mg/kg) every three days for 18 days. The tumor volumes were determined at the indicated time points by measuring tumor length (l) and width (w) and calculating tumor volume (V = 0.5 × l × w^2^). At the end of the experiment, the mice were killed after being anaesthetized by intraperitoneal injection of pentobarbital sodium (50 mg·kg−1), and the tumors were isolated by surgery in a room separated from the other animals. Then, the tumors were removed and weighed for *in vitro* experiments. Samples were prepared for histology and protein assays.

### Malondialdehyde (MDA) Assay

Tumor samples from nude mice were homogenized. The tissue lysates were then centrifuged at 12,000 × g for 10 min at 4°C to collect the supernatants. Total protein content was determined by the Bradford assay. MDA levels were detected using a Lipid Peroxidation MDA assay kit (Beyotime Institute of Biotechnology).

### Patient Samples

This study was approved by the Institutional Research Human Ethical Committee of Wenzhou Medical University for the use of clinical biopsy specimens, and informed consent was obtained from the patients. A total of 16 liver cancer biopsy samples from patients who were clinically diagnosed at the Fifth Affiliated Hospital of Wenzhou Medical University from 2015 to 2017 were analyzed. HCC tissues and matched tumor-adjacent morphologically normal liver tissues were frozen and stored in liquid nitrogen until further use.

### Immunohistochemistry and Haematoxylin and Eosin (H&E) Staining

Collected tumor tissues were fixed in 10% formalin at room temperature, processed and embedded in paraffin. Paraffin-embedded tissues were sectioned at 5 μm. After being hydrated, the tissue sections were incubated with primary antibodies overnight. Conjugated secondary antibodies and diaminobenzidine (DAB) were used for detection. Routine H&E staining was performed on mouse liver, kidney, and heart tissues. Sectional images were obtained with Image-Pro Plus 6.0 (Media Cybernetics, Inc., Bethesda, MD).

### Statistical Analysis

All experiments were carried out as three independent replicates (n = 3). The data are expressed as the means ± S.E.M.s. All statistical analyses were conducted using GraphPad Prism version 5.0 (GraphPad, San Diego, CA, USA). Student’s t-test was employed to analyze the differences between sets of data. A p-value < 0.05 indicated statistical significance.

## Results

### PL Increases ROS Levels and Significantly Inhibits the Proliferation of HCC Cells

To detect the effect of PL on HCC cells, we selected two HCC cells lines (HUH-7 and HepG2), treated them with increasing concentrations of PL for 24 h and evaluated cell viability using the MTT assay. PL treatment significantly decreased the viability of the two cell lines in a dose-dependent manner (**Figure 1B**). Next, we evaluated whether the killing effect of PL on HCC cells was related to ROS accumulation. ROS levels in HUH-7 cells were examined by flow cytometry using the redox-sensitive fluorescent probe 2’-,7’dichlorofluoresce in diacetate (DCFH-DA). PL treatment caused a time-dependent and dose-dependent increase in ROS levels in HUH-7 cell, which suggested that PL could disturb the levels of intracellular ROS. Interestingly, pretreatment with NAC, a specific ROS inhibitor, for 2 h apparently suppressed PL-induced increases in ROS levels (**Figures 1C, D**). Similarly, we detected the fluorescence intensity by a fluorescence microscope also discovered that PL may increase the levels of intracellular ROS and that this effect was almost completely reversed by pretreatment of the cells with NAC (**Figure 1E**). In addition, colony formation by HCC cells was significantly reduced when the cells were treated with PL. However, NAC fully abolished this reduction in colony formation induced by PL (**Figure 1F**). These results suggest that PL can induce ROS accumulation and cell death in HCC cells.

### PL Induces ROS-Dependent Apoptosis in HCC Cells

**Figure 2:**
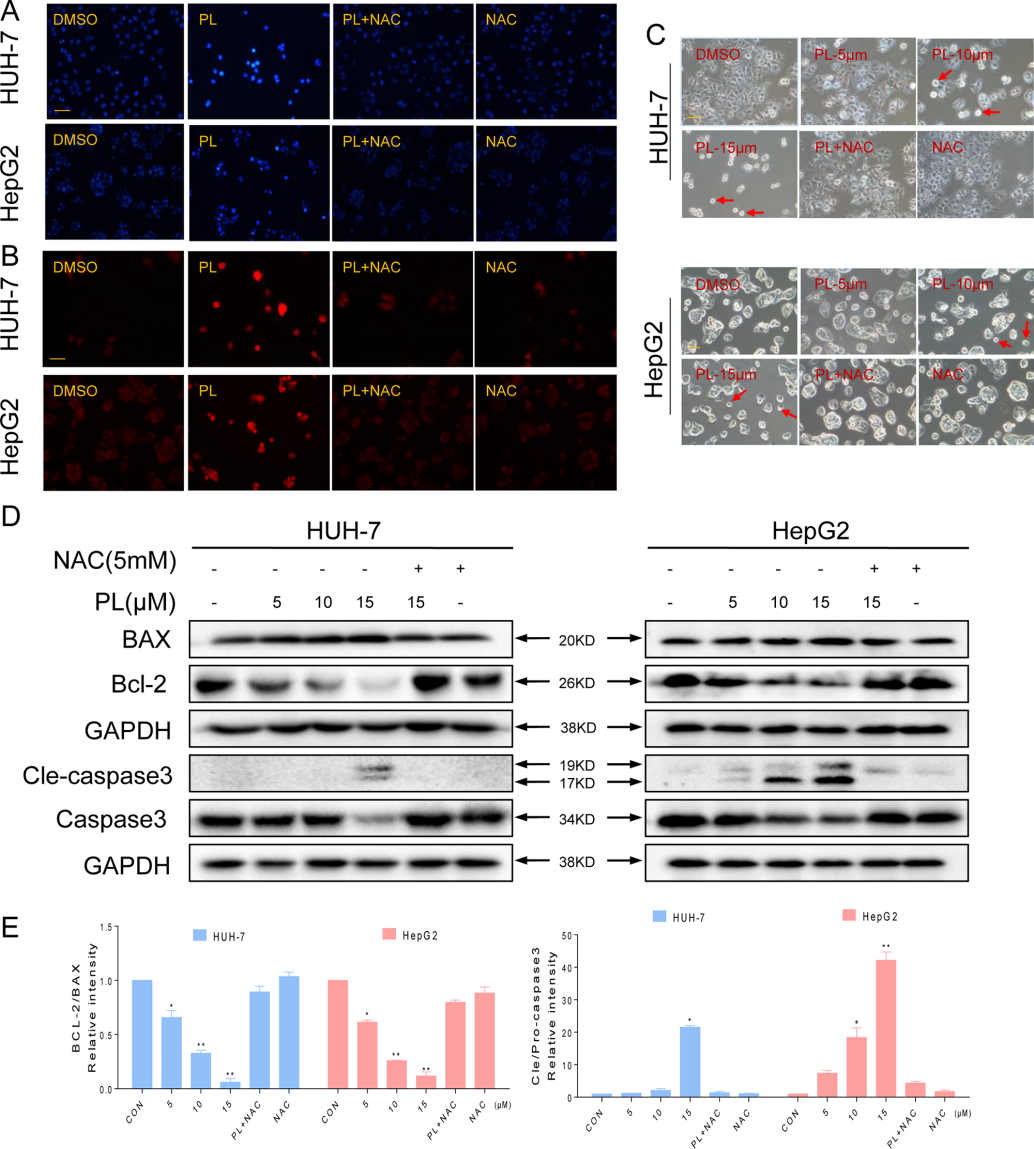
PL-induced apoptosis is dependent on intracellular ROS generation in HCC cells. **(A–C)** PL treatment induces apoptotic characteristics in HCC cells. Magnification, 200×. Bar, 100 µm. HCC cells were preincubated with or without 5 mM NAC for 2 h before exposure to PL (15 μM) for 24 h. Cell morphology was observed using an inverted microscope after Hoechst and PI staining. **(D)** Two HCC cell lines were preincubated with or without 5 mM NAC for 2 h before exposure to PL at the indicated concentration for 24 h, and apoptosis-related protein expression was determined by western blotting. Data represent similar results from three independent experiments. **(E)** Western blot results from **(D)** were calculated and compared with the BAX or caspase3. Western blot results were calculated and represent the percentage of the control (*p < 0.05, **p < 0.01). All images shown here are representative of three independent experiments with similar results.

To investigate the proapoptotic effects of PL in HCC cells, the two HCC cell lines were treated with PL in the presence or absence of NAC using Hoechst and propidium iodide (PI) staining assays. HCC cells exhibited the apoptotic characteristics nuclear condensation and fragmentation after treatment with PL for 24 h. NAC pretreatment almost completely reversed PL-induced apoptosis in HCC cells (**Figures 2A, B**). HCC cell apoptosis was also observed in PL-treated cells through morphological changes. The morphology of HCC cells changed markedly in comparison with the morphology of regular cancer cells. As observed under a microscope, the cancer cells became round and clearly shriveled following PL treatment. Pretreatment with NAC reversed the morphological changes in the cells induced by PL (**Figure 2C**). The proapoptotic effect of PL on HCC cells was further examined using a western blot assay. PL treatment decreased the levels of the antiapoptotic proteins Bcl-2 and procaspase3 and increased the levels of the proapoptotic proteins Bax and cleaved caspase-3 in a dose-dependent manner. Preincubation with NAC almost completely reversed these changes (**Figures 2D, E**). To conclude, these results confirmed that ROS induction mediates PL activated apoptotic pathways and is a vital upstream regulator of apoptosis.

### PL Induces ROS-Dependent G2/M Cell Cycle Arrest in HCC Cells

**Figure 3:**
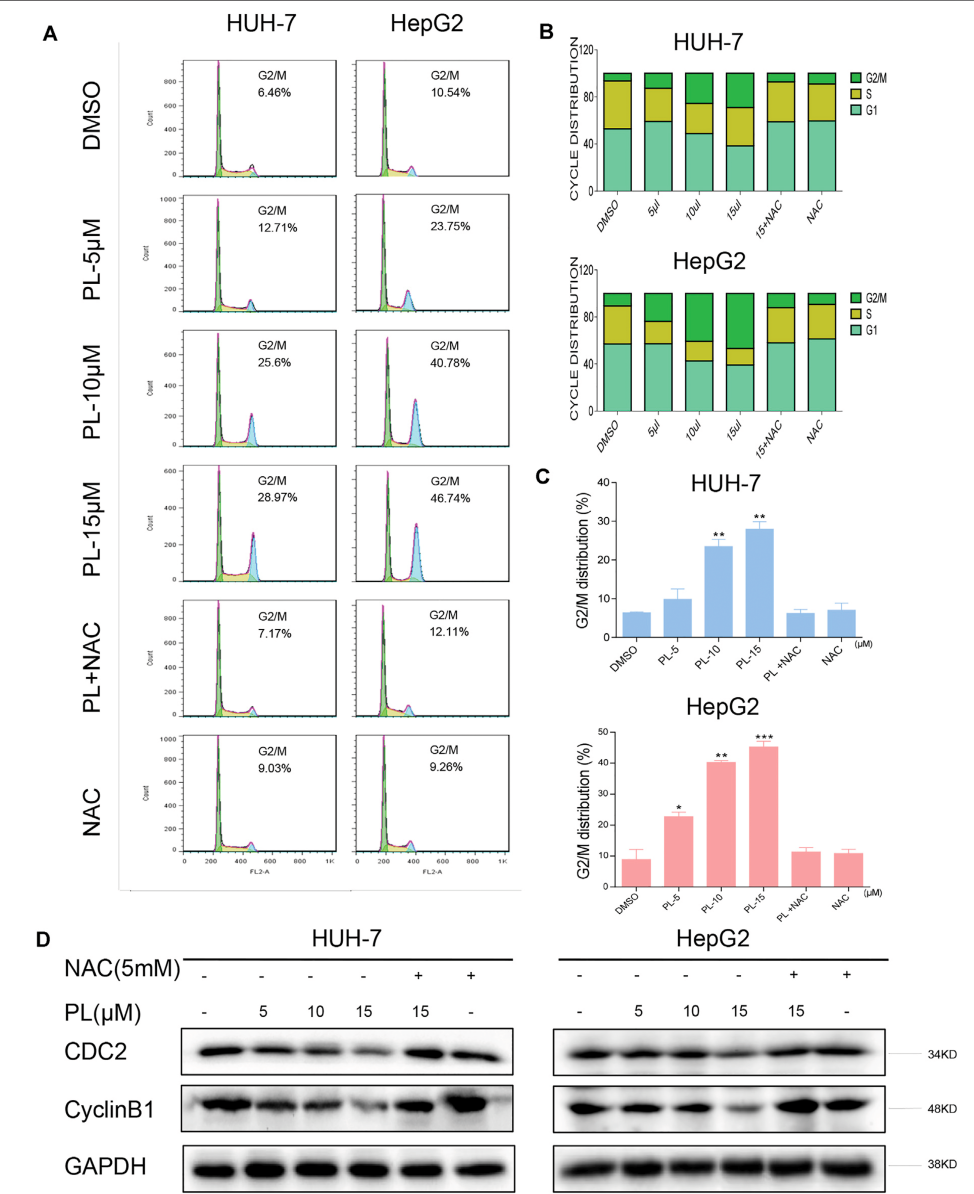
PL induces -induced cell cycle arrest is dependent on intracellular ROS generation in HCC cells. **(A)** HUH-7 and HepG2 cells were preincubated with or without 5 mM NAC for 2 h before exposure to PL at the indicated concentrations for 16 h. The cell cycle distribution was analyzed by flow cytometry. **(B** and **C)** Representative histogram from the cell cycle analysis shown in panel **(A)**. **(D)** Expression of G2/M phase-related proteins CyclinB1 and CDC2 in HCC cells exposed to the indicated concentration of PL with or without NAC (5 mM) for 20 h. GAPDH was used as an internal control. Data represent similar results from three independent experiments. Error bars represent the S.E.M. of triplicate experiments (*p < 0.05, **p < 0.01).

To confirm whether the growth inhibition in HCC cells by PL treatment was caused by cell cycle arrest, HCC cells were preincubated with NAC for 2 h before their exposure to various concentrations of PL for 16 h, and the cell cycle was then determined by flow cytometry. PL induced the accumulation of cells in G2/M phase in a dose-dependent manner, while the blocking of ROS generation by NAC completely attenuated PL-induced cell cycle arrest in HCC cells (**Figures 3A–C**). These flow cytometry data were mirrored by western blot analysis of cell cycle related proteins such as Cyclin B1 and Cdc2 in HCC cells (**Figure 3D**). These results revealed that the potent growth-inhibitory properties of PL are partly related to the induction of G2/M phase arrest and that ROS induction also mediates PL-induced G2/M phase cell cycle arrest.

### PL Activates ROS-Dependent ER Stress Signaling in HCC Cells

**Figure 4:**
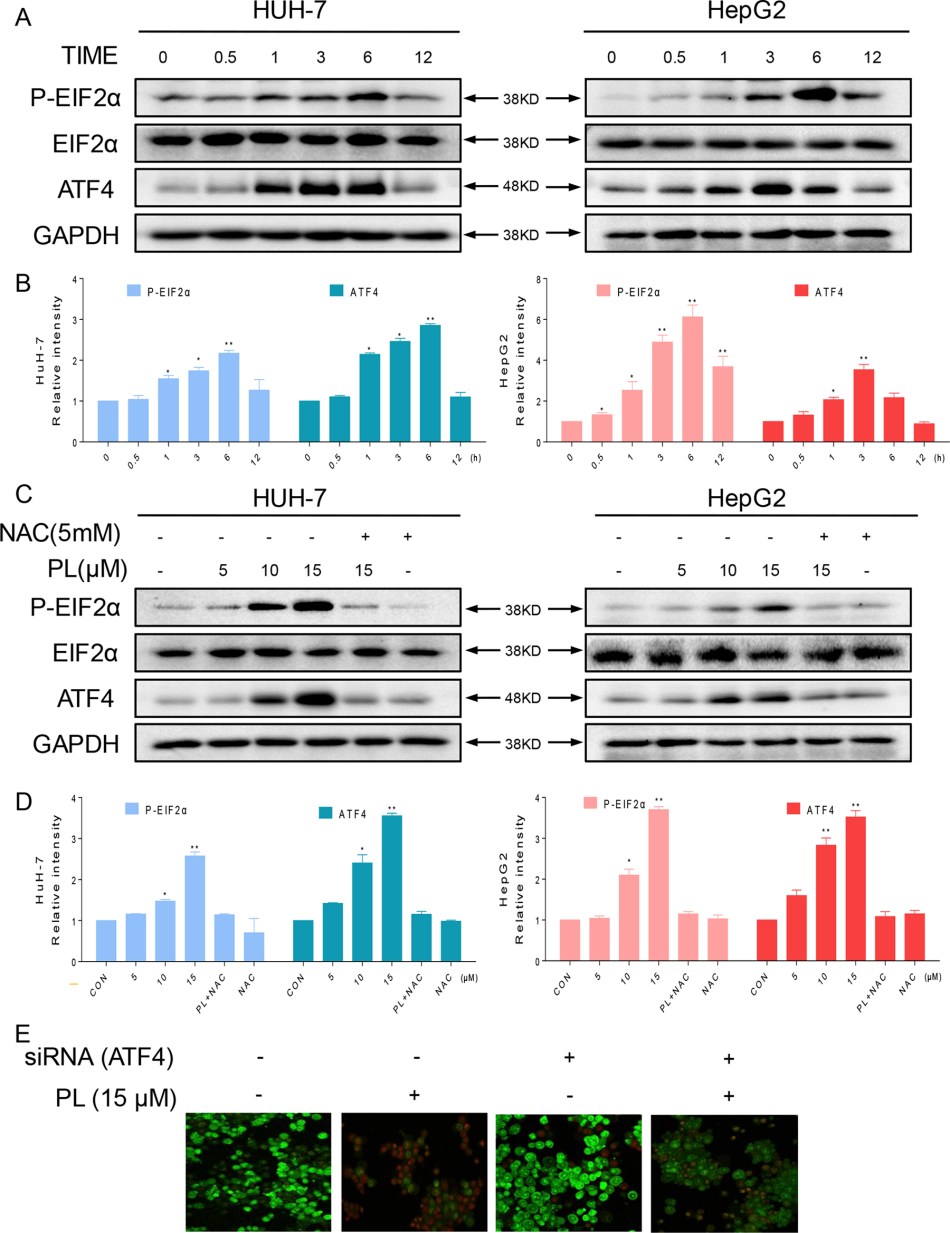
The ER stress pathway is involved in PL-induced apoptosis by promoting the accumulation of ROS. **(A)** HUH-7 and HepG2 cells were treated with PL (15 μM) for the indicated times, and the protein levels of p-eIF2α and ATF4 were determined by western blotting. GAPDH and eIF2α were used as internal controls. **(B)** Western blot results from **(A)** were calculated and compared with the control. **(C)** HUH-7 and HepG2 cells were pretreated with or without 5 mM NAC for 2 h before exposure to PL at the indicated concentrations. Six hours later, ATF4 and p-EIF2α expression was detected by western blot. GAPDH and eIF2α were used as internal controls. **(D)** Western blot results from **(C)** were calculated and compared with the control. **(E)** HUH-7 cells were transfected with siRNA against ATF4. Cells were then exposed to 15 μM PL and apoptotic cells were determined by acridine orange and ethidium bromide dual staining. Data represent similar results from three independent experiments. Western blot results were calculated and represent the percentage of the control (*p < 0.05, **p < 0.01).

As reported, ROS accumulation and redox status perturbation disrupt protein folding in the ER, causing ER stress (Plackova et al., 2016). Therefore, we attempted to understand whether PL induced ROS-dependent apoptosis was associated with ER stress. We examined the ER stress-related proteins, phosphorylated protein kinase RNA-like eukaryotic initiation factor 2α (p-EIF2α) and activating transcription factor-4 (ATF4), in PL-treated HCC cells. We recorded a time-dependent increase in ATF4 and phosphorylated eIF2α in HCC cells. Peak ATF4 and phosphorylated eIF2α levels were observed 3–6 h after the treatment of HCC cells with PL (**Figures 4A, B**). Furthermore, PL increased the expression of p-PERK and ATF4 in a dose-dependent manner. Importantly, pretreatment with the antioxidant NAC completely blocked the expression of these proteins in HCC cells (**Figures 4C, D**). Finally, to confirm if ER stress plays an essential role in response to PL-induced cell death, ATF4 was silenced in HUH-7 cells. ATF4-silencing led to significantly reduced number of apoptotic cells upon 15 µM PL treatment (**Figure 4E**). These findings indicated that the ER stress pathway may potentially be involved in PL-induced HCC cell apoptosis and that ROS induction also mediates PL-induced ER stress.

### TrxR1 Is Upregulated in HCC

**Figure 5:**
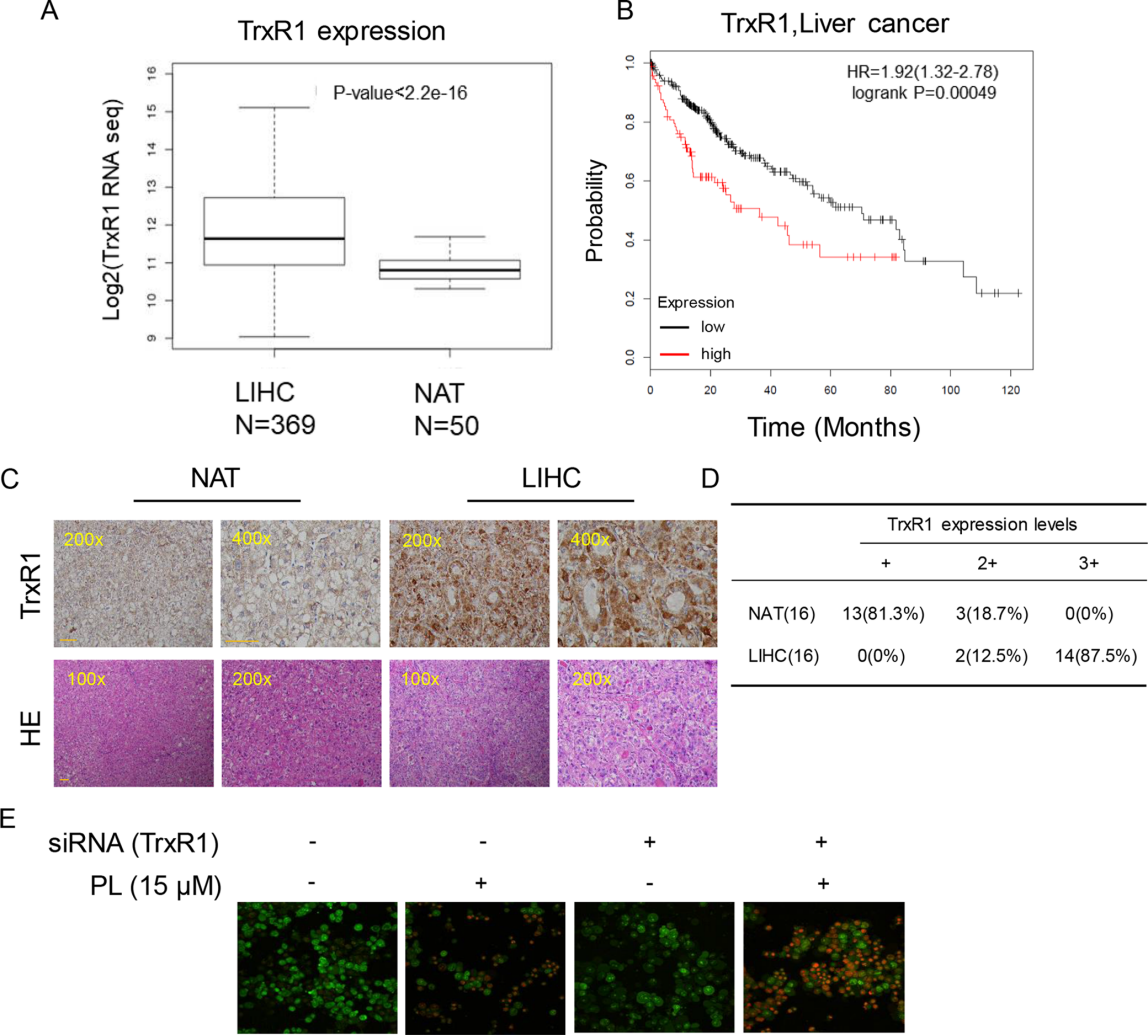
Upregulation of TrxR1 expression in LIHC. **(A)** TrxR1 levels in LIHC liver hepatocellular carcinoma and NATs normal adjacent tissues. **(B)** Higher increased TrxR1 protein expression predicts decreased survival. **(C)** Representative immunohistochemical staining for TrxR1 in LIHC and NATs. Bar, 100 µm. **(D)** Summary of immunohistochemical staining results. **(E)** HUH-7 cells transfected with TrxR1 siRNA and treated with 15 µM PL. Apoptotic cells were determined by acridine orange and ethidium bromide dual staining. Three independent experiments were performed.

Elevated levels of TrxR1 have been found in several malignancies and may be connected with aggressive tumor growth and poor survival. We speculated that TrxR1 is also overexpressed in HCC. Subsequent analyses using 369 liver hepatocellular carcinoma (LIHC) cases and 50 normal adjacent tissues (NATs) from the GSE59590 data set suggested that TrxR1 is significantly upregulated in LIHC tissues compared with its level in NATs (**Figure 5A**). Kaplan-Meier survival analysis showed that high TrxR1 expression is significantly correlated with poor patient survival (**Figure 5B**). Histopathological analyses were conducted to further examine TrxR1 expression in clinical LIHC. Analyzing of 16 evaluable paired clinical LIHC tissues and NATs, in which TrxR1 was measured in cancer tissues and compared with TrxR1 in corresponding NATs, revealed that TrxR1 was overexpressed in clinical LIHC cases (**Figures 5C, D**). These results supported the idea that TrxR1 is significantly upregulated in clinical LIHC tissues. In addition, to examine whether inhibition of TrxR1 was involved in PL-induced liver cancer apoptosis, we silenced TrxR1 in cells and exposed the cells to 15 µM PL. TrxR1 silencing significantly enhanced PL-induced HUH-7 cell apoptosis when compared to PL alone treated cells (**Figure 5E**). Thus, our findings indicates that PL directly targeted TrxR1 and induced apoptotic cell death by reducing TrxR1 activity.

### PL Inhibits HUH-7 Xenograft Tumor Growth Accompanied by Increased ROS Levels and Decreased Trxr1 Activity

**Figure 6:**
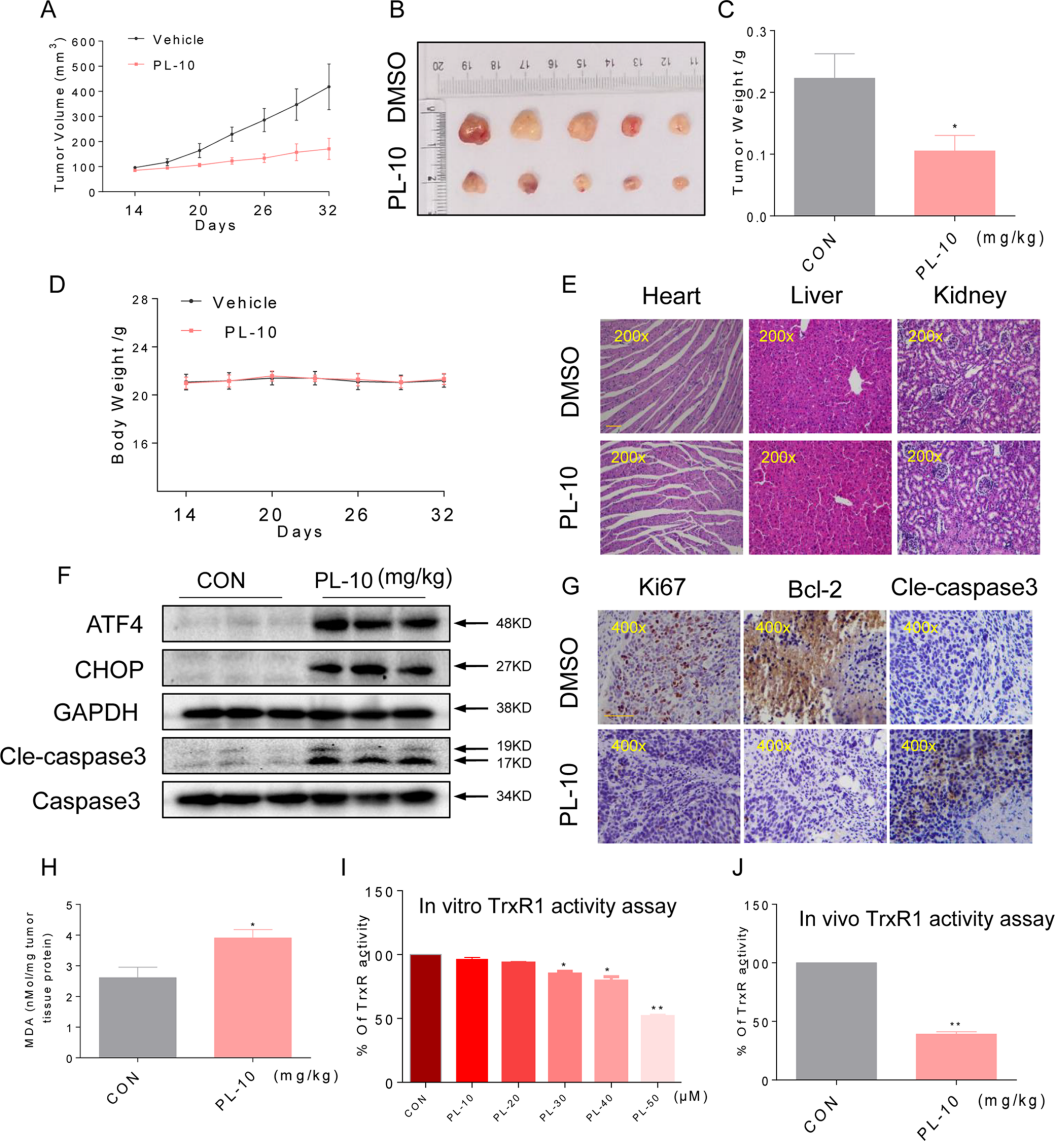
PL inhibits HUH-7 xenograft tumor growth accompanied by increasing ROS levels and decreasing TrxR1 activity. PL treatment inhibited tumor volume **(A–B)** and tumor weight. **(C)** of HUH-7 HCC xenografts in nude mice, but did not affect the body weight. **(D)** of the mice. **(E)** H&E staining images of kidney, liver, and heart tissues from the two groups showing no significant alterations. Bar, 100 µm. **(F)** Western blot analysis of ATF4, CHOP, and cleaved caspase-3 levels in resected tumor specimens. Bar, 100 µm. GAPDH and caspase-3 were used as loading control. **(G)** Immunohistochemical staining of tumor specimens for the cell proliferation marker Ki-67, the apoptosis marker cleaved caspase-3 and Bcl-2. **(H)** Levels of the oxidative stress marker MDA in the tumor tissues. **(I)** TrxR1 enzyme activity was measured with/without PL treatment *in vitro*. **(J)** TrxR1 activity of TrxR1 in tumor tissue lysates as determined by an endpoint insulin reduction assay. Data represent similar results from three independent experiments. Error bars represent the S.E.M. of triplicate experiments (*p < 0.05, **p < 0.01).

To assess the effect of PL treatment *in vivo*, we used a subcutaneous xenograft model of HUH-7 cells in immunodeficient mice. Next, We treated mouse HUH-7 tumors with PL. Treatment with 10 mg/kg PL for 18 days resulted in both a visual reduction in tumor volume and a reduction in tumor weight (**Figures 6A–C**). Importantly, no significant changes in body weight were observed in PL-treated mice compared to untreated mice (**Figure 6D**). We next examined vital organs (heart, liver, and kidney) to ascertain the any potential toxicity of PL and found that PL treatment is nontoxic (**Figure 6E**).

To determine whether the mechanisms we identified in our *in vitro* studies are also relevant to a xenograft model as vitro studies, we assessed the levels of key proteins identified from our culture studies. Western blotting analyses of the tumor tissues suggested that PL treatment increased the levels of ATF4, CHOP, and cleaved caspase-3 (**Figure 6F**), suggesting that PL-induced apoptosis in HUH-7 cells is connected to ER stress *in vivo*. Apoptosis, as assessed by the cleaved caspase-3 level, was increased in tumors following treatment with PL (**Figure 6G**). Correspondingly, PL treatment decreased the level of Bcl-2 and Ki-67 immunoreactivity. These findings indicated increased apoptosis and reduced cell proliferation in tumor tissues (**Figure 6G**). Moreover, PL treatment increased the levels of the product of lipid peroxidation (MDA) in tumor tissues (**Figure 6H**), suggesting increased ROS levels. In addition, TrxR1 activity in HUH-7 cells and tumor xenografts was measured by an endpoint insulin reduction assay, which showed that treatment with PL significantly reduced the activity of TrxR1(**Figures 6I, J**). In conclusion, these results support the targeting of TrxR1 by PL, which elevates oxidative stress and subsequently induces apoptosis in HCC.

## Discussion

Natural products have played an important role as effective sources of antitumor agents (Khoogar et al., 2016; Zhou et al., 2016). PL, a natural product isolated from the fruit of the long pepper, is a promising bioactive agent with proven antineoplastic effects on some tumor models (Wang et al., 2015). Relative to healthy cells, cancer cells harbor higher levels of ROS and exhibit an increased antioxidant defense system in an uncontrolled status (Trachootham et al., 2009; Denicola et al., 2011). As a result, cancer cells fail to deal with excrescent oxidative stress and become vulnerable to superfluous ROS (Raj et al., 2011; Glasauer and Chandel, 2014). This fact makes prooxidant cancer therapy an interesting area of study. Our results showed that PL could interfere with intracellular ROS levels in HCC cells, but a specific ROS inhibitor, NAC, significantly inhibited this PL-induced increase in ROS levels. Apoptosis usually manifests as cell contraction and separation, as well as nuclear condensation and fragmentation (Taatjes et al., 2008). By Hoechst, and PI staining using an inverted microscope, we observed that PL could induce ROS-dependent apoptosis. In addition, mitochondria are central to the regulation of apoptosis (Briehl et al., 2014). Several Bcl-2-family proteins, both antiapoptotic (Bcl-2) and proapoptotic (Bax), have C-terminal transmembrane domains that inserted in the outer membranes of mitochondria (Yip and Reed, 2008; Bhat et al., 2017). In this study, consistent with the observed morphological changes, treatment with PL significantly decreased the Bcl-2/Bax protein ratio in HCC cells. Importantly, NAC almost completely reversed these PL-induced changes in HCC cells. Imbalance of Bcl-2 family expression eventually leads to the apoptosis of HUH-7 and HepG2 cells. Thus, our findings revealed that ROS are pivotal upstream regulators of the anticancer activity of PL.

The ER is a crucial organelle in protein folding, modification, and secretion (Liu et al., 2015b). ER malfunction induced by various factors can lead to the unfolded protein response (UPR), resulting in ER stress. The UPR induces PERK-mediated phosphorylation of eIF2α, which attenuates normal mRNA translation but allows the preferential translation of ATF4 (Lafleur et al., 2013; Bhat et al., 2017; Marciniak, 2017; Oakes, 2017). ATF4 is a pivotal transcription factor in the ER stress pathway that mediates the induction of death-promoting transcriptional regulatory genes (Iurlaro and Munoz-Pinedo, 2016). As expected, PL was capable of inducing ROS-dependent ER stress in HCC cells, which led to cell death. In addition, the extent of ER stress following PL treatment was impaired after ROS were blocked by NAC, indicating that down-stream signaling was mediated by upstream signaling from ROS.

Redox homeostasis, the balance of which is maintained by two major cellular antioxidant systems, including the glutathione system and the thioredoxin system, is crucial for cellular viability and normal cellular functions (Benhar et al., 2016; Dagnell et al., 2018). Inside cells, the Trx system also acts as a redox regulator, which protects cells from damage caused by oxidative stress, scavenges ROS, and controls cellular redox balance (Koharyova and Kollarova, 2015). However, acting as a double-edged sword, TrxR1 both prevents and promotes cancer (Hatfield et al., 2009; Mahmood et al., 2013; Arner, 2017). In normal cells, TrxR1 can protect against oxidant stress and regulate cell apoptosis, whereas in tumor cells with high TrxR1expression (Du et al., 2012), the antiapoptotic function of TrxR1 promotes their growth and progression (Tonissen and Di Trapani, 2009). Moreover, Trx/TrxR system confers an aggressive tumor phenotype, poorer prognosis, decreased patient survival and resistance to programmed cell death (Mollbrink et al., 2014; Fu et al., 2017; Cho et al., 2019). Consistent with this, our results demonstrate that TrxR1 is overexpressed in LIHC and that high TrxR1 expression is associated with poor patient survival. This renders TrxR1 an interesting candidate for liver cancer chemotherapy. In this work, PL inhibited the enzyme function of TrxR1 and further shifted TrxR1 to an NADPH oxidase to generate superoxide anions, leading to ROS accumulation and ultimately eliciting oxidative stress.

**Figure 7:**
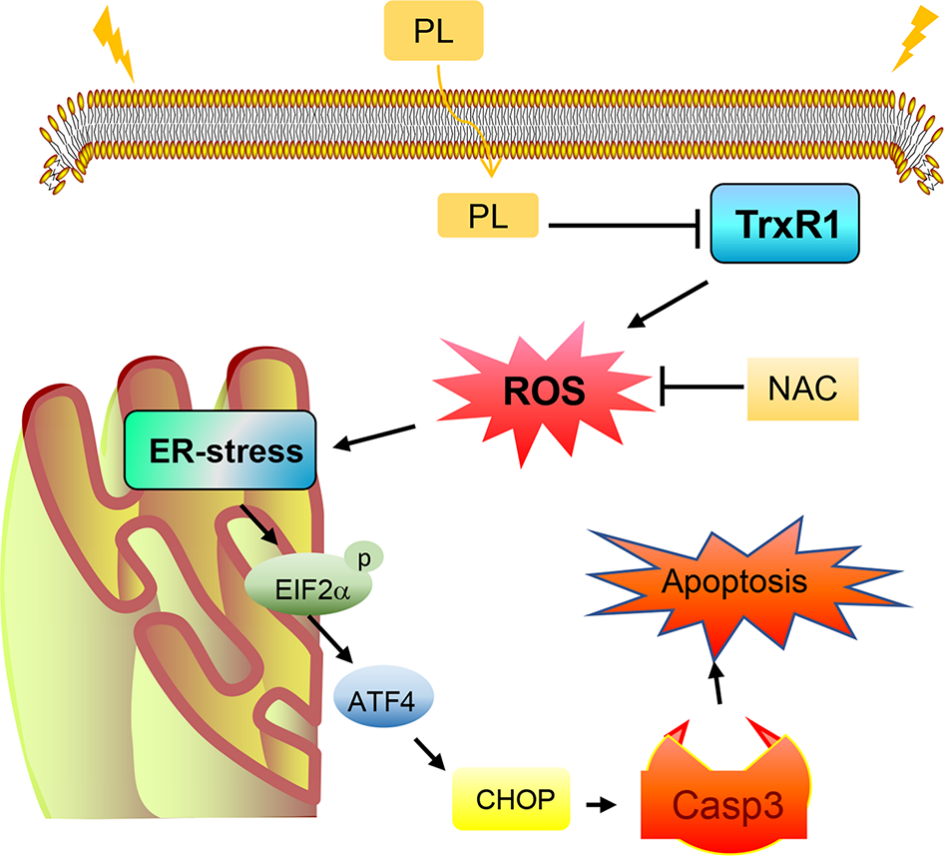
Schematic illustration of the underlying mechanism of the anticancer activity of PL.

We have summarized the possible mechanisms involved in PL-induced cell death in HCC based on analysis of the experimental results (**Figure 7**). In summary, we suggest TrxR1 as a novel target for liver cancer treatment and have demonstrated that PL induces ROS-dependent apoptosis in HCC cells by targeting TrxR1. Elucidating the PL-TrxR1 interaction may shed light on how this alkaloid acts *in vivo*, and understanding this novel targeting mechanism could lead to the development of small molecule inhibitors of TrxR1 as potential HCC chemotherapeutic agents.

## Data Availability Statement

The datasets generated for this study are available on request to the corresponding author.

## Ethics Statement

The animal study was reviewed and approved by Wenzhou Medical University.

## Author Contributions

QZ: Collection, analysis, and interpretation of data, manuscript writing. QZ, WC, XL, QW, MC, RC: collection and interpretation of data. GL, JJ: conception and design, interpretation of data, manuscript revision. All authors approved final version of the manuscript.

## Funding

The present study was supported by the National Natural Science Foundation of China (81573657 to JJ), the Key Research and Development Project of Zhejiang Province (2018C0302 to JJ) and the Zhejiang Province Medical and Health Care Key Project (2016146810 to JJ).

## Conflict of Interest

The authors declare that the research was conducted in the absence of any commercial or financial relationships that could be construed as a potential conflict of interest.

## Abbreviations

ATF4, activating transcription factor 4; Bax, associated protein x; Bcl-2, Bcell lymphoma 2; Cdc2, cyclin-dependent kinase 1 (cell division cycle protein 2); DCFH-DA, 2′, 7′-dichlorodihydrofluorescin diacetate; eIF2, eukaryotic initiation factor 2; ER, endoplasmic reticulum; HRP, horseradish peroxidase; Ki-67, nuclear protein associated with cell proliferation; MDA, malondialdehyde; MTT, 3-(4,5-dimethylthiazol-2-yl)-2,5diphenyltetrazolium bromide; NAC, N-acetyl cysteine; PI, propidium iodide; ROS, reactive oxygen species.
