# Corrigendum: Piperlongumine, a Novel TrxR1 Inhibitor, Induces Apoptosis in Hepatocellular Carcinoma Cells by ROS-Mediated ER Stress

**DOI:** 10.3389/fphar.2022.806724

**Published:** 2022-07-08

**Authors:** Qianqian Zhang, Weiqian Chen, Xiuling Lv, Qiaoyou Weng, Minjiang Chen, Ri Cui, Guang Liang, Jiansong Ji

**Affiliations:** ^1^ Key Laboratory of Imaging Diagnosis and Minimally Invasive Intervention Research, the Fifth Affiliated Hospital of Wenzhou Medical University/Affiliated Lishui Hospital of Zhejiang University/Lishui Central Hospital, Lishui, China; ^2^ Chemical Biology Research Center, School of Pharmaceutical Sciences, Wenzhou Medical University, Wenzhou, China

**Keywords:** thioredoxin reductase 1, reactive oxygen species, hepatocellular carcinoma, endoplasmic reticular stress, piperlongumine

In the original article, there was a mistake in “ Figure 3D” as published. “we unintentionally used the same GAPDH images in the two figures (the GAPDH band below Bcl-2 in Figure 2D is the same one as in the Figure 3D which is below the CyclinB1 in both cells)”. The corrected “Figure 3” appears below.

**FIGURE 3:**
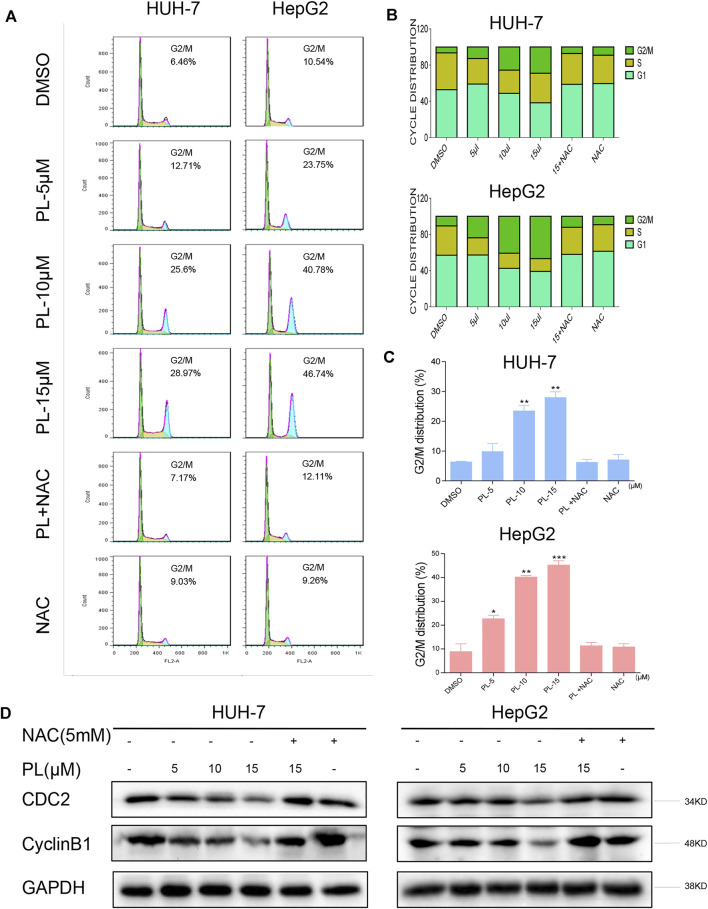
PL induces -induced cell cycle arrest is dependent on intracellular ROS generation in HCC cells. **(A)** HUH-7 and HepG2 cells were preincubated with or without 5 mM NAC for 2 h before exposure to PL at the indicated concentrations for 16 h. The cell cycle distribution was analyzed by flow cytometry. **(B and C)** Representative histogram from the cell cycle analysis shown in panel **(A)**. **(D)** Expression of G2/M phase-related proteins CyclinB1 and CDC2 in HCC cells exposed to the indicated concentration of PL with or without NAC (5 mM) for 20 h. GAPDH was used as an internal control. Data represent similar results from three independent experiments. Error bars represent the S.E.M. of triplicate experiments (*p < 0.05, **p < 0.01).

The authors apologize for this error and state that this does not change the scientific conclusions of the article in any way. The original article has been updated.

